# Antimicrobial resistance, virulence associated genes and phylogenetic background versus plasmid replicon types: the possible associations in avian pathogenic *Escherichia coli* (APEC)

**DOI:** 10.1186/s12917-022-03496-x

**Published:** 2022-11-30

**Authors:** Maad Tohmaz, Mahdi Askari Badouei, Hamideh Kalateh Rahmani, Gholamreza Hashemi Tabar

**Affiliations:** Department of Pathobiology, Faculty of Veterinary Medicine, Ferdowsi University of Mashhad, Mashhad, Iran

**Keywords:** *Escherichia coli*, APEC, AMR, Phylotype, Inc., Plasmid, Replicon typing

## Abstract

**Background:**

Antimicrobial resistance (AMR) in bacterial isolates from food producing animals not only challenges the preventive and therapeutic strategies in veterinary medicine, but also threatens public health. Genetic elements placed on both chromosome and plasmids could be involved in AMR. In the present study, the associations of genomic backbone and plasmids with AMR were evaluated. We also provided some primary evidences that which genetic lineages potentially host certain groups of plasmids.

**Results:**

In the current study, 72 avian pathogenic *Escherichia coli* (APEC) strains were examined. Isolates resistant to tetracycline and trimethoprim-sulfamethoxazole (87.5%; each), and harboring *bla*_TEM_ (61.1%) were dominant. Moreover, phylogroup D was the most prevalent phylogroup in total (23.6%), and among multidrug-resistant (MDR) isolates (14/63). The most prevalent Inc-types were also defined as follows: IncP (65.2%), IncI1 (58.3%), and IncF group (54.1%). Significant associations among phylogroups and AMR were observed such as group C to neomycin (*p* = 0.002), gentamicin (*p* = 0.017) and florfenicol (*p* = 0.036). Furthermore, group D was associated with *bla*_CTX_. In terms of associations among Inc-types and AMR, resistance to aminoglycoside antibiotics was considerably linked with IncP (*p* = 0.012), IncI1 (*p* = 0.038) and IncA/C (*p* = 0.005). The *bla*_TEM_ and *bla*_CTX_ genes presence were connected with IncI1 (*p* = 0.003) and IncFIC (*p* = 0.013), respectively. It was also shown that members of the D phylogroup frequently occured in replicon types FIC (8/20), P (13/47), I1 (13/42), HI2 (5/14) and L/M (3/3).

**Conclusions:**

Accorging to the results, it seems that group D strains have a great potential to host a variety of plasmids (Inc-types) carrying different AMR genes. Thus, based on the results of the current study, phyogroup D could be a potential challenge in dealing with AMR in poultry. There were more strong correlations among Inc-types and AMR compared to phylotypes and AMR. It is suggested that in epidemiological studies on AMR both genomic backbone and major plasmid types should be investigated.

**Supplementary Information:**

The online version contains supplementary material available at 10.1186/s12917-022-03496-x.

## Background

Colibacillosis, an important poultry bacterial infection caused by Avian Pathogenic *Escherichia coli* (APEC), is responsible for major losses in the poultry industry worldwide. Despite the fact that it has been identified for more than a century, poultry colibacillosis remains as one of the most important poultry diseases that leads to mortality, reduction in productivity, and economic losses [1]. Unlike intestinal *Escherichia coli* pathotypes, APEC members mainly cause extraintestinal infections in poultry and the pathogenicity of these strains has not been clearly elucidated. In most studies, APEC pathogenicity has been attributed to the presence and expression of different virulence factors such as: surface antigens, fimbriae, intimin, colicin, heat-sensitive hemagglutinin, iron acquisition systems, serum resistance, toxins and etc. [2–4]. Since there is no single genetic determinant to identify APEC, efforts have been made to identify minimal predictors for diagnostic purposes. Accordingly, panel of five genes including: *iroN* (salmochelin), *iutA* or *aerJ* (aerobactin), *ompT* (outer membrane protease), *iss* (serum resistance) and *hlyF* (toxin) has been widely accepted for APEC virulotyping [5, 6].

Therapeutic, metaphylactic and prophylactic purposes are the main reasons of wide application of antibiotics in the poultry industry. The antibiotics usage in poultry farms around the world does not follow a single pattern. However, tetracyclines, macrolides, sulfonamides and aminoglycosides are recorded as the most common prescribed antibiotics in different regions [7–9]. The misuse of antibiotics has been contributed to emerging and spread of antimicrobial resistance (AMR), and evolution of multidrug-resistant (MDR) pathogens resulting in poor treatment and public health concerns. Genetic poential of resistance to antibiotics may acquire by mutation or horizontal gene transfer (HGT) [10]. Plasmids carrying AMR genes are effective transmitters of this feature in microbial populations which are preserved and propagated under the selective pressure resulting from the indiscriminate use of antibiotics [11, 12]. It is belived that plasmid-mediated AMR transfer has a key role in increasing the population of MDR strains among *Enterobacterales* [13]. As an example, common presence of mobile genetic elements responsible for resistance to β-lactam antibiotics, tetracyclines and sulfonamides in APEC has been recorded [14, 15]. Resistance to β-lactam antibiotics due to the production of extended-spectrum beta-lactamase (ESBL) would be one of the most concerning AMR which leads to inactivation of a broad spectrum of antibiotics such as: penicillins, third and fourth generation of cephalosporins, and monobactam [16]. Moreover, it has been suggested that poultry industry acts as a reservoir for ESBL-producing *E. coli* [17].

Considering the importance of plasmids in bacterial biology, the identification and classification of plasmids can be highly informative. The first method proposed for classification of plasmids used the plasmid incompatibility phenomenon [18]. Actually, plasmid incompatibility (Inc) is defined as the inability of two dependent plasmids to survive for a long time in a common cell [19]. After that, a PCR-based method for classifying plasmids has been developed, known as PBRT (PCR-Based Replicon Typing). This method is able to identify the main plasmid families in *Enterobacterales* [20]. It has been shown that certain plasmids are related to transferring specific antibiotic resistance to particular bacterial clones like *Escherichia coli* ST131 clade C2/*H*30Rx and IncFII plasmids carrying *bla*_CTX-M_ [10]. It should be noted that other traits such as: virulence, enhanced fitness and metabolism of rare substances could also be acquired through plasmids [21].

The effect of genetic background in acquisition of antibiotic resistance has also been described. While some members of phylogenetic groups A and D are prone to acquire resistance against third-generation cephalosporins, B2 strains are more susceptible [22]. The most common method for identifying phylogroups is a multiplex PCR which is able to categorize *Escherichia coli* isolates into eight phylogenetic groups (A, B1, C, E, D, F, B2 and E. Clades). Importantly, phylotyping is a valuable method because it is believed that *E. coli* strains are not randomly distributed throughout the bacterial populations. Members of same group tend to have similar characteristics in pathogenicity, niche, and resistance [23]. Although a vast majority of research has been investigated AMR along with phylotypes, the information about AMR relation to easily spreadable plasmid types has been overlooked.

In the present study, 72 APEC strains which have been isolated from poultry in recent years, were examined in terms of virulence-associated genes (VAG), plasmid (Inc) types, phylogenetic lineages and drug resistance (phenotypic AMR and ESBL genes). The results of this study can lead to a better understanding of the distribution and importance of genomic backbone and plasmid content in APEC strains isolated from poultry and its relationship with the spreading patterns of drug resistance.

## Results

### Antibiotic susceptibility testing

A total of 72 *Escherichia coli* isolates were investigated for susceptibility to ten antibiotics of eight categories (tetracycline, trimethoprim-sulfamethoxazole, amoxicillin-clavulanic acid, neomycin, florfenicol, enrofloxacin, gentamicin, cefazolin, colistin and furazolidone) by agar-disk diffusion method. According to the results of antibiogram test, the highest rates of antimicrobial resistance were observed against tetracycline (63/72; 87.5%) and trimethoprim-sulfamethoxazole (63/72; 87.5%), followed by enrofloxacin (59/72; 81.94%) and florfenicol (41/72; 56.94%). In addition, the lowest rates of bacterial resistance belonged to colistin (0/72, 0%) and cefazolin (3/72, 4.17%). Moreover, 63 isolates (87.5%) were considered MDR strains based on being resistant to three or more classes of antibiotics.

**Fig. 1:**
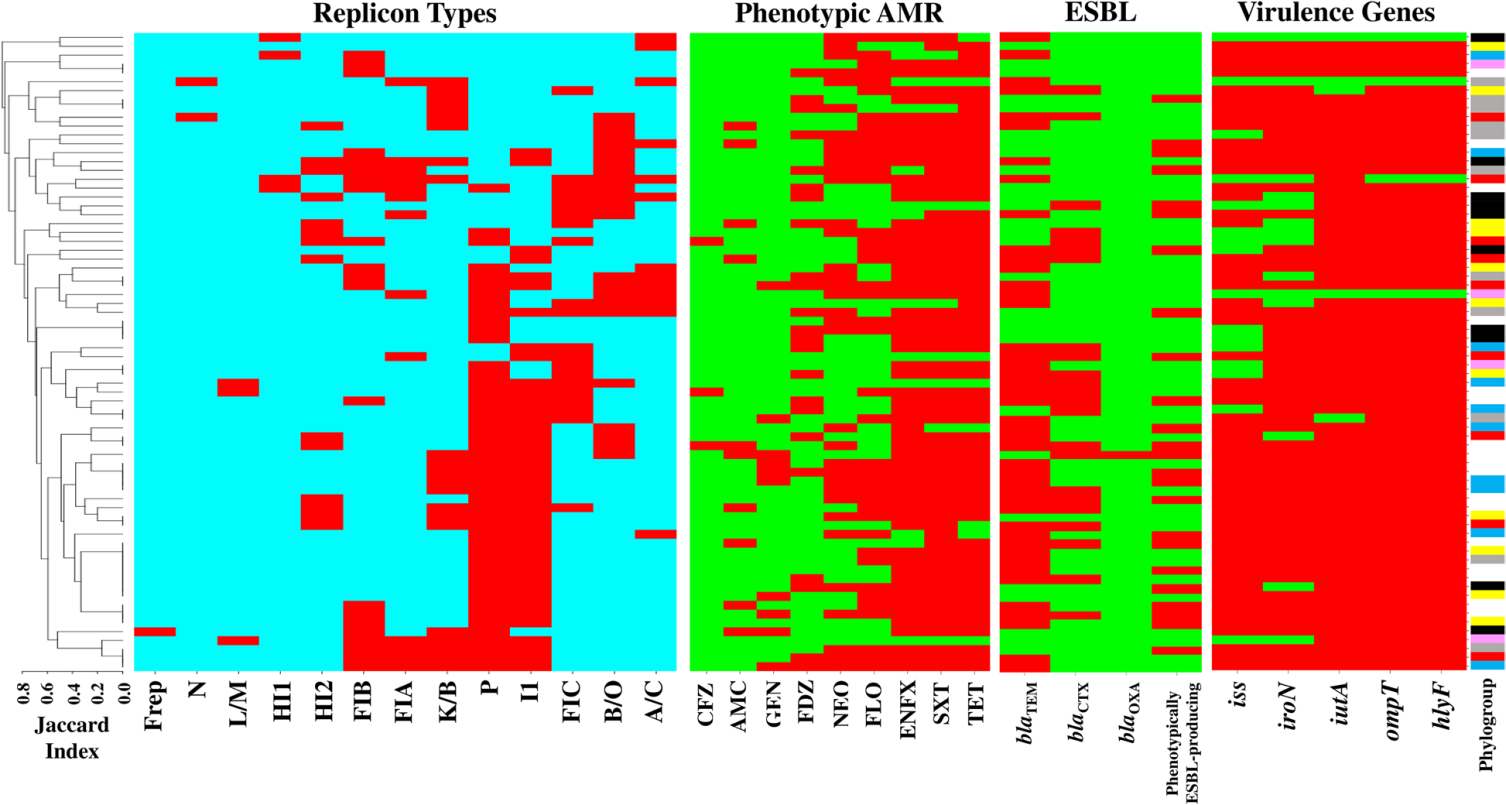
Heatmap. Genotypes and phenotypes of the APEC strains. Red: presence of genetic elements/not susceptible to antibiotics; Blue: absence of replicon types; Green: absence of genes/susceptible to antibiotics. Phylogroups: A=yellow; B1=red; B2=pink; C=black; D=white; E=light blue; F=gray.  Cluster analysis was carried out based on the replicon types

Molecular detection of ESBL-producing *E. coli*, revealed the presence of *bla*_TEM_, *bla*_CTX_ and *bla*_OXA_ genes alone or in combination with each other in 49 (68.05%) strains. The prevalences of genes are as follows: *bla*_TEM_ (44/72; 61.1%), *bla*_CTX_ (23/72; 31.94%) and *bla*_OXA_ (1/72; 1.38%). No isolate was detected positive for *bla*_SHV_. Moreover, 24 (33.33%) strains were phenotypically confirmed as ESBL-producing strains. Table 1 and Fig. 1, represents the results in details.

**Table 1:** Characteristics of the APEC isolates examined in the current study including phylogroup, virulence genes, Inc-types, AMR and ESBL (phenotype and genotype)

No.	Phylogroup	Virulence genes	Inc-type	AMR/ESBL^**a**^	ESBL genes
**Virulence score 0**					
6	A	*–*	I1-A/C-HI1	NEO,FLO,ENFX,SXT	*bla*_TEM_
24	A		K/B-A/C-FIA-N	NEO,FLO	*bla*_TEM_
27	A		P-B/O-A/C-FIA	NEO,FLO,ENFX,SXT,TET	*bla*_TEM_
**Virulence score 1**					
7	B1	*iutA*	B/O-K/B-FIB-FIC-A/C-FIA-HI1	NEO,FLO,ENFX,SXT,TET	*bla*_TEM_
**Virulence score 3**					
50	D	*ompT, hlyF, iutA*	P-I1-K/B-FIB-FIA-L/M	–	–
58	D		P-HI2	NEO,FLO,ENFX,SXT,TET/ESBL	*bla*_CTX_
68	F		P-FIB-FIC-HI2	FLO,ENFX,SXT,TET,CFZ/ESBL	*bla*_CTX_
69	F		B/O-FIC	−/ESBL	*bla*_CTX_
**Virulence score 4**					
12	A	*ompT, hlyF, iss, iutA*	P-I1-B/O-FIB-A/C	NEO,ENFX,SXT,TET,FDZ	–
19	A		P-I1-B/O-HI2	ENFX,SXT,TET,FDZ	*bla*_TEM_
3	B2		P-I1	NEO,FLO,ENFX,SXT,TET,FDZ	–
2	F		P-B/O-FIC-A/C	TET	*bla*_TEM_
23	F		B/O-FIC-HI2-A/C-FIA	ENFX,SXT,TET,FDZ	–
59	F		FIC-HI2	NEO,ENFX,SXT,TET,FDZ,AMC	–
10	B1	*iroN, ompT, hlyF, iutA*	B/O	NEO,FLO,ENFX,SXT,TET,FDZ	–
29	B1		P	NEO,FLO,ENFX,SXT,TET,FDZ	–
55	B1		P	ENFX,SXT,TET,FDZ	–
71	B1		P-FIC	ENFX,SXT,TET/ESBL	*bla*_TEM_
72	B1		P-FIC	ENFX,SXT,TET,FDZ	*bla*_CTX_, *bla*_TEM_
45	D		I1	FLO,ENFX,SXT,TET/ESBL	*bla*_CTX_, *bla*_TEM_
52	D		I1-FIC	ENFX,SXT,TET,FDZ/ESBL	*bla*_CTX_, *bla*_TEM_
54	D		P-I1-FIC	ENFX,SXT,TET,FDZ	*bla*_CTX_
51	D	*iroN, ompT, hlyF, iss*	P-I1-FIC	FLO,ENFX,SXT,TET,GEN/ESBL	*bla*_TEM_
66	D		K/B-FIC	FLO,ENFX,SXT,TET/ESBL	*bla*_CTX_, *bla*_TEM_
**Virulence score 5**					
14	A	*iroN, ompT, hlyF, iss, iutA*	P-FIB-A/C	NEO,ENFX,SXT,TET	*bla*_TEM_
25	A		P-I1	FLO,ENFX,SXT,TET,GEN	–
26	A		P-I1-K/B-HI2	NEO,FLO,ENFX,SXT,TET	–
35	A		I1-B/O-K/B-FIB-HI2-FIA	NEO,FLO,ENFX,SXT,TET	*bla*_TEM_
62	A		B/O-K/B-N	FLO,ENFX,SXT,TET/ESBL	*bla*_CTX_, *bla*_TEM_
65	A		B/O-K/B-HI2	FLO,ENFX,SXT,TET,AMC	*bla*_TEM_
4	B1		B/O-FIC-FIA	SXT,TET	*bla*_TEM_
38	B1		K/B	ENFX,SXT,TET,FDZ	–
41	B1		P-K/B-FIB-Frep	ENFX,SXT,TET,AMC,GEN	–
5	B2		FIB-HI1	NEO,FLO,TET	*bla*_TEM_
18	B2		A/C	NEO,SXT,TET	–
36	B2		FIB	FLO,ENFX,SXT,TET	–
1	C		P-I1-BO	NEO,ENFX/ESBL	*bla*_TEM_
	C		P-I1-B/O-FIB-A/C	NEO,FLO,ENFX,SXT,TET,FDZ,GEN/ESBL	*bla*_TEM_
11	C		P-I1-A/C	NEO,FLO,SXT/ESBL	*bla*_TEM_
13	C		I1-B/O-FIB	NEO,FLO,ENFX,SXT,TET	–
16	C		P-I1-K/B-FIB-FIA	NEO,FLO,ENFX,SXT,TET,FDZ	*bla*_TEM_
17	C		P-I1-K/B-FIB-FIA	NEO,FLO,ENFX,SXT,TET,FDZ,GEN	*bla*_TEM_
20	C		P-I1-K/B	NEO,FLO,ENFX,SXT,TET,GEN/ESBL	*bla*_TEM_
28	C		P-I1-K/B	NEO,FLO,ENFX,SXT,TET	*bla*_CTX_, *bla*_TEM_
31	C		P-I1-K/B	NEO,FLO,ENFX,SXT,TET,FDZ,GEN/ESBL	*bla*_TEM_
33	C		P-I1-K/B-FIB-FIA	NEO,FLO,ENFX,SXT,TET	–
48	D		P-I1-B/O-FIC-L/M	–	*bla*_CTX_, *bla*_TEM_
49	D		P-I1-FIC-L/M	FLO,ENFX,SXT,TET,CFZ/ESBL	*bla*_CTX_, *bla*_TEM_
53	D		P-I1-FIB-FIC	ENFX,SXT,TET,FDZ/ESBL	*bla*_CTX_, *bla*_TEM_
56	D		P-I1	ENFX,SXT,TET,FDZ	*bla*_CTX_, *bla*_TEM_
57	D		B/O-A/C	NEO,FLO,ENFX,SXT,TET,AMC	–
60	D		P-I1-B/O-HI2	NEO,ENFX,SXT,TET,AMC,CFZ/ESBL	*bla*_CTX_, *bla*_TEM_
6	D		P-I1-HI2	NEO,FLO,ENFX,SXT,TET/ESBL	*bla*_CTX_, *bla*_TEM_
63	D		P-I1-K/B-FIC-HI2	FLO,ENFX,SXT,TET,AMC/ESBL	*bla*_CTX_, *bla*_TEM_
67	D		P-I1-K/B-HI2	ENFX,SXT	*bla*_CTX_, *bla*_TEM_
70	D		P	NEO,FLO,ENFX,SXT,TET	–
21	E		FIB	NEO,FLO,ENFX,SXT,TET,FDZ	–
22	E		P-I1-FIB	NEO,FLO,ENFX,SXT,TET,GEN	*bla*_CTX_, *bla*_TEM_
34	E		K/B	NEO,TET,FDZ	–
37	E		P-I1-B/O-K/B	ENFX,SXT,TET,AMC,GEN/ESBL	*bla*_CTX_, *bla*_OXA_
39	E		P-I1	ENFX,SXT,TET/ESBL	*bla*_TEM_
40	E		P-I1-FIB	ENFX,SXT,TET/ESBL	*bla*_TEM_
42	E		P-I1-FIB	FLO,ENFX,SXT,TET,AMC	*bla*_TEM_
43	E		P-I1	FLO,ENFX,SXT,TET/ESBL	*bla*_TEM_
44	E		P-I1	FLO,ENFX,SXT,TET	*bla*_TEM_
47	E		P-I1	SXT,TET,AMC	*bla*_CTX_, *bla*_TEM_
9	F		P-B/O-FIB-FIC-FIA-HI1	ENFX,SXT,TET,FDZ	–
15	F		P-I1-B/O-FIC-A/C	NEO,ENFX,SXT,TET,FDZ	–
30	F		B/O-FIB-HI2-FIA	NEO,FLO,SXT,TET,FDZ	–
32	F		P-I1-K/B	NEO,FLO,ENFX,SXT,TET,GEN/ESBL	*bla*_TEM_
46	F		I1-FIC-FIA	−/ESBL	*bla*_CTX_, *bla*_TEM_
64	F		I1-HI2	FLO,ENFX,SXT,TET,AMC	*bla*_CTX_, *bla*_TEM_

^a^phenotypically ESBL-producing isolates

### Phylogenetic groups

The 72 analyzed APEC strains were classified into seven phylogenetic groups: A, B1, B2, C, D, E and F. Group D was the most prevalent phylogenetic group (17; 23.61%) while only few strains defined as members of group B2 (4; 5.55%). The distribution of remaining strains in other groups are as follows: A (11; 15.27%), F (11; 15.27%), C (10; 13.88%), E (10; 13.88%), and B1 (9; 12.5%).

### APEC virulence-associated genes and virulence score

The five genes *iroN*, *ompT*, *hlyF*, *iss* and *iutA* are considered as minimal predictos of APEC virulence-associated genes (VAG) [5]. In the current study, the virulotyping of the isolates were carried out which resulted in seven patterns. Isolates were also scored based on the number of VAG possesed. The virulence scores (VS) of the strains were in the range of 0 to 5. The most prevalent VS was 5 (48/72; 66.66%). Moreover, the most prevalent VAGs were *ompT* (68/72; 94.4%) and *hlyF* (68/72; 94.4%). The prevalences of the genes *iutA*, *iroN* and *iss* were defined 67 (93.05%), 58 (80.55%) and 56 (77.77%), respectively.

### Plasmid replicon typing

The applied PBRT method is able to identify 18 plasmid replicon types. In the present study, only 13 replicon types were detected in the investigated APEC strains. All isolates possessed at least one typable replicon. Furthermore, plasmid replicon type P was the most frequent type among strains (47/72; 65.28%). The second frequent plasmid replicon type was I1 (42/72; 58.33%) followed by B/O (22/72; 30.56%), FIB and K/B (21/72; 29.17% each), whilst the plasmid replicons T, W, FIIA, Y and X were absent. In addition to frequencies of individual replicons, investigation of the presence of IncF group (FIA, FIB, FIC, FIIA, Frep) are important. More than half of the isolates (39/72; 54.16%) benefited from having one or combination of the members of IncF group. The profiles of IncF group among the isolates are as follows: FIA (2/39); FIB (10/39); FIC (13/39); FIA-FIB (6/39); FIA-FIC (3/39); FIB-FIC (2/39); FIB-Frep (1/39); and FIA-FIB-FIC (2/39).

Moreover, 50 different patterns of plasmid replicon combinations were observed which is represented in Fig. 1. The richest pattern belonged to a B1 member with simultanious presence of seven (B/O-K/B-FIB-FIC-A/C-FIA-HI1) replicons. Moreover, the pattern P-I1 was the most prevalent pattern (7/72; 9.72%).

### Distribution of phenotypic AMR and ESBL (phenotype and genotype) among phylogenetic groups

Isolates resistant to trimethoprim-sulfamethoxazole, tetracycline, enrofloxacin, florfenicol, neomycin and furazolidone were found in all phylogroups, while resistant isolates to gentamicin, amoxicillin-clavulanic acid and cefazolin were detected in certain phylogenetic groups. Moreover, no notable assosiation was observed among MDR and phylogroups.

Overall, the phylogenetic group D was the most resistant phylotype. Resistance to nine antibiotics was detected in this group (all the tested antibiotics except colistin). Members of D also had the most participation in resistance to enrofloxacin (15/59; 25.42%), cefazolin (2/3; 66.7%), tetracycline (14/63; 22.2%) and trimethoprim-sulfamethoxazole (15/63; 23.8%). Actually, resistance to cefazolin were observed only in D and F groups. Moreover, groups B2 and C had no resistant isolates to amoxicillin-clavulanic acid as well. The results of AMR distribution among phylogenetic groups are represented in details in Table 2.

**Table 2:** Prevalence of AMR and ESBL (phenotype and genotype) among phylogenetic groups of 72 APEC isolates (n; % within each phylogroup)

Phylogroup (n)	Phenotypic AMR										β-Lactamase/ESBL			
	SXT	TET	ENFX	FLO	NEO	FDZ	GEN	AMC	CFZ	MDR	Phenotype	***bla***_**TEM**_	***bla***_**CTX**_	***bla***_**OXA**_
**A (11)**	10 (90.9)	9 (81.81)	10 (90.9)	8 (72.72)	7 (63.63)	2 (18.18)	1 (9.09)	1 (9.09)	0	10 (90.9)	1 (9.09)	8 (72.72)	1 (9.09)	0
**B1 (9)**	9 (100)	9 (100)	8 (88.88)	3 (33.33)	3 (33.33)	5 (55.55)	1 (11.11)	1 (11.11)	0	8 (88.88)	1 (11.11)	4 (44.44)	1 (11.11)	0
**B2 (4)**	3 (75)	4 (100)	2 (50)	3 (75)	3 (75)	1 (25)	0	0	0	4 (100)	0	1 (25)	0	0
**C (10)**	9 (90)	8 (80)	9 (90)	**9**^**P**^**(90)**	**10**^**P**^**(100)**	4 (40)	**4**^**P**^**(40)**	0	0	9 (90)	4 (40)	8 (80)	1 (10)	0
**D (17)**	15 (88.23)	14 (82.35)	15 (88.23)	9 (52.94)	5 (29.41)	4 (23.52)	1 (5.88)	3 (17.64)	2 (11.76)	14 (82.35)	**10**^**P**^**(58.82)**	12 (70.58)	**13**^**P**^**(76.47)**	0
**E (10)**	9 (90)	10 (100)	8 (80)	5 (50)	3 (30)	2 (20)	2 (20)	3 (30)	0	10 (100)	4 (40)	7 (70)	3 (30)	1 (10)
**F (11)**	8 (72.72)	9 (81.81)	7 (63.63)	4 (36.36)	4 (36.36)	5 (45.45)	1 (9.09)	2 (18.18)	1 (9.09)	8 (72.72)	4 (36.36)	4 (36.36)	4 (36.36)	0
**Total (72)**	63 (87.5)	63 (87.5)	59 (81.9)	41 (56.9)	35 (48.6)	23 (31.94)	10 (13.9)	10 (13.9)	3 (4.2)	63 (87.5)	24 (33.33)	44 (61.11)	23 (31.94)	1 (1.38)
***p*-value**	0.637	0.562	0.315	0.110	**0.006**	0.447	0.234	0.535	0.539	0.570	0.055	0.164	**0.000**	0.392

^*P*^ Positive correlation

^*^Bold numbers showed significant association (*p* < 0.05)

From ten antibiotics, only the distribution of neomycin (*p* = 0.006) showed significant difference in relation to different phylogroups. Other noticeable results are the considerable correlations between group C and resistance to neomycin (10/35; 28.57%; *p* = 0.002), gentamicin (4/10; 40%; *p* = 0.017) and florfenicol (9/41; 21.95%; *p* = 0.036).

From three ESBL genes (*bla*_TEM_, *bla*_CTX_, *bla*_OXA_) detected in the current study, only *bla*_CTX_ showed significant difference (*p* = 0.000) in distribution among phylogenetic groups. The gene *bla*_CTX_ (*p* = 0.000) and phenotypically ESBL-producing strains (*p* = 0.018) were also considerably related to phylogroup D. For correlation coefficients see Supplementary 1.

### Distribution of phenotypic AMR isolates and ESBL (phenotype and genotype) among plasmid replicon types

Isolates resistant to enrofloxacin, tetracycline and trimethoprim-sulfamethoxazole were distributed among all the replicon types, while resistance to gentamicin, cefazolin, amoxicillin-clavulanic acid, neomycin, florfenicol and furazolidone were related to some Inc-types. Members of IncP were the most prevalent resistant isolates to all the tested antibiotics. Along with IncP, replicon type I1 had also the majority of resistent isolates to amoxicillin-clavulanic acid (6/10) and florfenicol (25/41). Table 3, represents distribution of AMR isolates among plasmid replicon types in details.

**Table 3:** Prevalence of phenotypic AMR and ESBL (phenotype and genotype) among plasmid replicon types of 72 APEC isolates (n)

Replicon (n)	Phenotypic AMR										β-Lactamase/ESBL			
	SXT (63)	TE (63)	ENF (59)	FLO (41)	NEO (35)	FDZ (23)	GEN (10)	AMC (10)	CFZ (3)	MDR (63)	Phenotype (24)	***bla***_**TEM**_ (44)	***bla***_**CTX**_ (23)	***bla***_**OXA**_ (1)
**B/O (22)**	18	19	17	10	12	8	2	4	1	17	5	12	5	1
**FIC (20)**	16	17	15	**6**^**N**^	**3**^**N**^	8	1	2	2	15	10	13	**11**^**P**^	0
**A/C (13)**	11	10	9	7	**11**^**P**^	4	1	1	0	11	2	8	**0**^**N**^	0
**P (47)**	43	42	**42**^**P**^	25	22	15	**10**^**P**^	6	3	42	18	31	16	1
**K/B (21)**	18	18	18	15	12	5	6	4	0	18	7	14	6	1
**FIA (13)**	10	10	**8**^**N**^	8	8	5	1	0	0	**9**^**N**^	**1**^**N**^	8	**1**^**N**^	0
**FIB (21)**	19	20	18	14	13	8	4	2	1	20	4	11	3	0
**I1 (42)**	38	35	37	25	20	12	**9**^**P**^	6	2	37	**18**^**P**^	**32**^**P**^	17	1
**Frep (1)**	1	1	1	0	0	0	1	1	0	1	0	0	0	0
**HI1 (4)**	3	3	3	3	3	1	0	0	0	4	0	3	0	0
**N (2)**	1	1	1	2	1	0	0	0	0	1	1	2	1	0
**HI2 (14)**	14	13	13	9	7	4	0	**5**^**P**^	2	13	5	8	7	0
**L/M (3)**	**1**^**N**^	**1**^**N**^	1	1	0	0	0	0	1	**1**^**N**^	1	2	2	0

Bold numbers showed significant association (*p* < 0.05)

^*P*^ Positive correlation

^*N*^ Negative correlation

In the current study, resistance to aminoglycoside antibiotics was considerably linked with IncP, IncI1 and IncA/C. Actually, replicon types P (*p* = 0.012) and I1 (*p* = 0.038) had significant presence in gentamicin resistance, and IncA/C (*p* = 0.005) significantly reflected resistance to neomycin. Moreover, statistical analysis clarified other significant relations (*p* < 0.05) among replicon types and antibiotic resistance. The IncHI2 were considerably prevalent (*p* = 0.020) among resistant isolates to amoxicillin-clavulanic acid. In contrast to positive relations, there were significant associations and negative correlations between isolates possessed IncL/M, IncFIC or IncFIM replicon types and some of the antibiotic resistance. These relations are as follows: IncL/M and tetracycline (*p* = 0.039), IncL/M and sulfamethoxazole-trimethoprim (*p* = 0.039), IncFIC and neomycin (*p* = 0.000), IncFIC and florfenicol (*p* = 0.007). The association between IncFIA and enrofloxacin was marginally significant (*p* = 0.050). Moreover, IncFIA (*p* = 0.050) and IncL/M (*p* = 0.039) were negatively related to MDR.

Significant relations among ESBL genes (*bla*_TEM_, *bla*_CTX_, *bla*_OXA_) and replicon types were detected. Genes *bla*_TEM_ and *bla*_CTX_ were positively connected with IncI1 (*p* = 0.003) and IncFIC (*p* = 0.013), respectively. On the other hand, *bla*_CTX_ had a tendency to participate in IncA/C (*p* = 0.006) and IncFIA (*p* = 0.050). Furthermore, phenotypically confirmed ESBL strains had notable associations with IncFIA (*p* = 0.048) and IncI1 (*p* = 0.048). For correlation coefficients see Supplementary file 1.

### Association of plasmid replicon types and phylogenetic groups

The replicon types P and FIB were distributed among all the detected phylogenetic groups. Members of phylogenetic group D were the most prevalent isolates in replicon types FIC (8/20), P (13/47), I1 (13/42), HI2 (5/14) and L/M (3/3).

It should be noted that statistical analysis revealed no significant difference in distribution of plasmid replicon types among phylogenetic groups; except for IncFIC (*p* = 0.000), IncI1 (*p* = 0.000) and IncHI2 (*p* = 0.015). Significant associations with positive or negative correlations were observed among replicons and phylogenetic groups. Memebers of F group had considerable tendency for replicon types FIC (8/11; 72.72%; *p* = 0.001) and HI2 (5/11; 45.45%; *p* = 0.032), while strains belonged to C were associated with I1 (10/10; 100%; *p* = 0.004) replicon. Moreover, high presence of IncA/C in group A (5/11; 45.45%; *p* = 0.022) and IncL/M in group D (3/17; 17.64%; *p* = 0.011) were observed. Table 4, presents distribution of plasmid replicon types among phylogroups in details. Furthermore, correlation coefficients are available in Supplementary 1.

**Table 4:** Prevalence of plasmid replicon types among phylogenetic groups of 72 APEC isolates (n)

Phylogroup n)	Plasmid replicon types (n)												
	B/O (22)	FIC (20)	A/C (13)	P (47)	K/B (21)	FIA (13)	FIB (21)	I1 (42)	Frep (1)	HI1 (4)	N (2)	HI2 (14)	L/M (3)
**A (11)**	6	**0**^**N**^	**5**^**P**^	6	5	3	3	6	0	1	**2**^**N**^	4	0
**B1 (9)**	3	4	1	5	3	2	2	**0**^**N**^	1	1	0	0	0
**B2 (4)**	0	0	1	1	0	0	2	1	0	1	0	0	0
**C (10)**	3	0	2	9	6	3	5	**10**^**P**^	0	0	0	0	0
**D (17)**	3	8	1	13	4	1	2	13	0	0	0	5	**3**^**P**^
**E (10)**	1	0	0	8	2	0	4	8	0	0	0	0	0
**F (11)**	6	**8**^**P**^	3	5	1	4	3	4	0	1	0	**5**^**P**^	0
***p*-value**	0.086	**0.000**	0.103	0.113	0.103	0.175	0.396	**0.000**	0.312	0.411	0.077	**0.015**	0.119

Bold numbers showed significant association (*p* < 0.05)

^*P*^ Positive correlation, ^*N*^ Negative correlation

### Relations among VAG, VS, AMR, ESBL genes, phylogroups, and replicons

Interestingly, most of the notable (*p* < 0.05) associations amog VAG and VS with AMR, ESBL genes, phylogroups and Inc-types were negatively correlated, except for *iss* and IncI1 (*p* = 0.020), VS and tetracycline (*p* = 0.003), and VS and FIB (*p* = 0.050). Other considerable relations and correlation coefficients are available in Supplementary 1.

## Discussion

Emergence of AMR isolates in food producing animals is not only a challenge to the veterinay preventive and therapeutic strategies, but also a serious public health concern. Genetic factors responsible for AMR to different classes of antibiotics are located on both transferable plasmids and chromosomes (in forms of transposons, integrons and genetic cassets), but plasmids have a pivotal role in HGT of AMR in bacterial populations especially in *Enterobacterales* [24]. Thus, plasmid contents and types are issues of interest which were mostly neglected in AMR studies.

In the present study, comparisons between genetic backbone (phylotypes), plasmid types (Inc-type) and virulence genotypes with AMR were made to evaluate which relates better to AMR. We also provided some primary evidences that which genetic lineages potentially host certain groups of plasmids. Since the genetic basis of resistance to different antimicrobials are quite diverse, we have chosen the phenotypic resistance to some classes of commonly prescribed antibiotics in animals and humans. Moreover, molecular investigation of common ESBL genes and VAG carried out to see if a notable associations could be find.

Various AMR profiles were observed in the current study and 87.5% of the strains were confirmed as MDR. Moreover, high resistance to tetracycline (63/72, 87.5%), sulfamethoxazole-trimethoprim (63/72, 87.5%) and enrofloxacin (59/72, 81.94%) was defined which is in line with other reports [25, 26]. The presence of larg number of MDR strains and resistant isolates to “critically important antimicrobials” such as enrofloxacin means that taking proper preventive and theraputic strategies based on using antimicrobial agents for colibacillosis needs special considerations, as there are limited available alternatives for “critically important antimicrobials” [27]. Therefore, emerging resistance to the mentioned antibiotics means more obstacles to cure infections in the forthcoming future. In the present study, more than half of the strains (49/72; 68.05%) carried at least one of the ESBL genes: *bla*_TEM_, *bla*_CTX_ and *bla*_OXA_. Interestingly, the prevalence of *bla*_TEM_ was almost two times greater than *bla*_CTX_ (44 compared to 23). This pattern of distribution of the ESBL genes is different from other studies [28, 29] as it has been shown that *bla*_CTX_ was the predominant lineage of ESBL genes for over the last two decades [30]. Moreover, in the present study, almost half of the ESBL gene carrying isolates were confirmed as phenotypically ESBL-producing strains. It should be noted that the importance of phenotypically negative ESBL strains has been showen before as strains with unexpressed ESBL genes also has a role in spreading of ESBL genes via HGT or resulting in ESBL phenotype back in certain conditions [31].

In the current study, 72 APEC strains were assigned into seven groups (A, B1, B2, C, D, E, F) with highest frequencies of phylogenetic groups D (17/72; 23.61%) followed by A (11/72; 15.3%) and F (11/72; 15.3%). While group A is related to human commensal strains [32], strains from phylogroup D are associated with human infections which its high prevalence among APEC isolates is concerning [23]. Since the zoonotic risk of chicken-source phylogroup F *E. coli* and its contribution to spread of MDR *E. coli* to humans heve been proposed recently [33, 34], the high portion of group F members should not be neglected. Moreover, the observed scattered distribution of APEC strains in different phylogroups may indirectly reflects the commensal nature of APEC strains who broken the host defence and ended up a septicemic infection [1].

In our study, only few significant associations were observed among phylogenetic groups and AMR. The most notable ones were related to the groups C and D. The group C strians had considerable relation to phenotypic resistant to neomycin (10/35; 28.57%; *p* = 0.002), gentamicin (4/10; 40%; *p* = 0.017) and florfenicol (9/41; 21.95%; *p* = 0.036), while group D was highly associated with *bla*_CTX_. Furthermore, in the present study, members of different phylotypes showed the same resistance patterns (R-types) and vice versa that indicates phylotyping might not be a robust genotyping strategy to predict AMR except for few antimicrobials. One of the possible explanation could be due to the very general bacterial classification in Clermont’s method based on some conserved chromosomal genes. Furthermore, plasmids which have a pivotal role in AMR are neglected in this typing method.

In the present study, variety of patterns for plasmid replicons (50 different profiles for 72 strains) were observed. Different AMR genes, virulence determinants and fitness factors could be coded by plasmids. Based on PBRT method applied in the present study, among 72 APEC strains, only 13 replicon types were observed. Out of them, IncP (47/72, 65.27%), IncI1 (42/72, 58.3%) and IncF group (FIA, FIB, FIC, FIIA, Frep; 39/72, 54.16%) were the most prevalent plasmid types. High prevalence of IncP among the isolates (47/72, 65.27%) differs the current study from previous reports on APEC, as they have recorded lower values or no presence of P replicon type [6, 35–37]. The IncP plasmids are composed of genes responsible for resistance to a wide variety of antibiotic classes, heavy metals and quaternary ammonium compounds in gram negative bacteria. It is believed that the selective pressure of antimicrobial agents or antiseptics in clinical conditions have resulted in spread and maintenance of IncP plasmids [38, 39]. Moreover, it has been demonstrated that IncI1 plasmid type are prevalent among *E. coli* of the avian hosts as our results support it too [35]. The high frequency of IncF group in commensals, environmental strains, APEC and other pathotypes of *E. coli* such as STEC, has been mentioned by others as well [35, 40, 41]. However, no presence of IncF group replicons was also reported in *bla*_*CMY-2*_ plasmids in MDR *E. coli* from poultry [42]. The significant aspects of the IncF group and I1 replicons are their association to virulence (such as fimbriae and type IV pili, respectively) and antibiotic resistance determinants (ESBL genes in particular) which are therapeutic concerns [19, 43].

Relations among plasmid replicon types and AMR have been investigated in numerous studies [19, 24]. The current study defined significant assosiations as well. Resistance to aminoglycosides was considerably linked with IncP, I1 and A/C replicon types which is also mentioned in other studies [24]. Importantly, plasmid replicon types P and A/C are broad-host-range plasmids which can disseminate the aminoglycoside resistance genes among different bacterial hosts [19]. Besides, significant association was observed between resistance to amoxicillin-clavulanic acid and plasmid replicon types HI2. One of the most resistance traits associated with HI2 replicon type is ESBL genes [19]. Along with HI2, different studies have mentioned the association of *bla*_CTX_ with IncF family, IncA/C, IncL/M replicon types, although the relation between FIC and *bla*_CTX_ was confirmed in our study [44] . Moreover, it has been proven that IncI1 is associated with several β-lactamases including *bla*_TEM_, which our results support it too [45]. Based on our results and other reports, it seems that in the cases of AMR surveys in APEC, it is not necessary to looking for every Inc-types introduced by Carattoli et al., [20], as some of the plasmid Inc-types (IncT, IncW, IncX) have been recorded in low prevalence or absent in most studies. On the other hand, detection of broad-host-range plasmids that could transfer AMR genes among different species like: IncP, IncA/C, IncI1 and IncH, and high prevalent replicon types such as IncI and IncF group would be essential in studies investigating antibiotic resistance, especially plasmid mediated AMR [36, 42, 46].

The replicon types P and FIB were distributed among all the detected phylogenetic groups. Plasmids belonging to the mentioned replicon types are conjugative plasmids which could be disseminated among bacterial hosts [24]. Moreover, members of phylogenetic group D were the most prevalent isolates in replicon types FIC (8/20), P (13/47), I1 (13/42), HI2 (5/14) and L/M (3/3). Accorging to this, it seems that group D strains have a great potential to host variety of plasmids belonging to different replicon types. Since IncP, IncI1, IncHI2 and IncL/M are associated with different antimicrobial resistance genes [24], it could be a possible explaination for high participation of D members in MDR group of strains (14/63; 22.2%). Associations of plasmid replicon types and phylogroups are not fully described so far. However, the present study revealed notable relations with positive and negative correlations. Significant high presence of IncFIC (8/11; 72.72%; *p* = 0.001) and IncHI2 (5/11; 45.45%; *p* = 0.032) in group F, IncI1 (10/10; 100%; *p* = 0.004) in group C, IncA/C in group A (5/11; 45.45%; *p* = 0.022), and IncL/M in group D (3/17; 17.64%; *p* = 0.011) were observed. Another important point is that almost all of the group C strains possess I1 and P replicons. In contrast to positive associations, negative relations were also defined. Members of group B1 significantly lacked IncI1 (0/9; *p* = 0.000) and group A isolates lacked IncFIC (0/11; *p* = 0.028) and N (2/11; 18.18%; *p* = 0.022). In fact, the mentioned associations are preliminarly data and more comprehensive studies are necessary to verify the associations between the genetic backbone (phylogroups) and plasmid contents. However, according to our results, it seems that phylogroups D and C are prone to aquire and share various plasmid mediated genes as they harbor broad-host-range plasmids such as: P, I1 and L/M replicon types.

## Conclusions

In the current study, APEC strains were defined as a heterogeneous population. Numerous variations were observed in gentotypic features (phylogenetic groups, virulence types, plasmid replicon types) and AMR (phenotypic and ESBL genes) of the isolates. Comparisons were conducted to evaluate the correlations among genetic criteria with AMR. The results also showed the weakness of phylogenetic grouping in prediction of AMR. Furthermore, it seems that in the cases of AMR surveys in APEC, not all of the plasmid replicon types are equally important. Some of the Inc-types like: IncP, IncI, IncL/M, IncA/C, IncH and IncF group have a higher priority due to their broad-host-range or their high prevalence. We believe that these types should be screened in most AMR studies to define the AMR spread with more clarity. Moreover, further studies are needed to clarify how genetic backbone and plasmid contents are affected by each other.

## Methods

### Bacterial strains

A panel of 72 non-duplicate *E. coli* strains used in the present study were randomly selected from microbial collection (Ferdowsi University of Mashhad, Mashhad, Iran). All the isolates had been recovered from clinical cases of avian colibacillosis (heart or liver samples) occurred in Iran. The identity of the strains were confirmed based on the colony morphology, bacterial morphology and gram staining, lactose fermentation in MacConkey agar, catalase and oxidase production, standard biochemical tests including IMViC (indole, methyl red, acetoin production, citrate utilization), triple sugar agar (TSI) and urease production [47]. To revive each isolate, 50 μl of cryopreserved bacteria were streaked on MacConkey agar to achieve a single colony. Then, a single colony was propagated on Luria-Bertani (LB) agar plates for the next steps.

### DNA extraction

DNA was extracted according to the boiling method. Briefly, a loop of bacteria from an overnight culture (18–20 hour) was suspend in 400 μl of sterile distilled water. The suspension was boiled in a boiling water bath for 10 min and after cooling on ice buckets, centrifuged at 800×g for 5 minutes. Finally, supernatant was collected and preserved as DNA template for further molecular investigations [1].

### Antimicrobial resistance

#### Phenotypic resistance

To evaluate susceptibility of APEC strains against different classes of antibiotics, standard agar-disk diffusion method (Kirby-Bauer) was performed [48]. All the isolates (*n* = 72) were screened for 10 antibiotics from eight classes of commonly used antibiotics in veterinary and human therapeutics. The antibiotic disks were as follow: amoxicillin-clavulanic acid (AMC 20/10 μg), tetracycline (TET 30 μg), neomycin (NEO 30 μg), florfenicol (FLO 30 μg), enrofloxacin (ENFX 5 μg), gentamicin (GM 10 μg), trimethoprim-sulfamethoxazole (SXT 1.25/23.75 μg), colistin (CST 10 μg), cefazolin (CFZ 30 μg) and furazolidone (FDZ 100 μg). Final results were achieved based on comparison of diameter of inhibited growth zones to CLSI interpretive criteria [49].

#### Phenotypic confirmatory disc diffusion test for ESBL production

According to the procedure recommended by CLSI, ceftazidime (CTZ 30 μg) and cefotaxime (CTX 30 μg) alone and with clavulanate (10 μg) were placed on Muller-Hinton plates inoculated with fresh bacterial suspension with a dilution equal to 0.5 McFarland. After 18 h incubation at 37 °C, inhibition zone was measured. ESBL-producer strain was confirmed when a ≥ 5-mm in a zone diameter for either antimicrobial agent tested in combination with clavulanate versus the zone diameter of the agent when tested alone was observed [49].

#### β- lactamase/ ESBL genes

Molecular detection of some widespread *β-* Lactamase /ESBL gene families of *E. coli* was carried out using a triplex PCR reaction for detection of *bla*_TEM_, *bla*_SHV_, *bla*_OXA_ and a uniplex PCR for detection of *bla*_CTX-M_. Each PCR reactions was performed in a volume of 20 μl containing: 10 μl *Taq* DNA Polymerase Master Mix RED 2x (Amplicon, Denmark) containing 1.5 mM MgCl_2_, various concentration of each primers, ultrapure water and 300 ng of template DNA. Primer characteristics and thermal conditions are shown in Table 5. Finally, PCR products were analyzed by electrophoresis using 1.5% (w/v) agarose gel and Green Viewer safe stain (0.01 v/v).

**Table 5:** Primers used in the present study

Panel	Primer pair	Sequence (5′ to 3′)	Annealing temp (°C)	Product size (bp)	Ref.
Phylogenetic grouping					
Quadruplex	*chuA*	F: ATGGTACCGGACGAACCAAC R: TGCCGCCAGTACCAAAGACA	59	288	[23]
	*yjaA*	F: CAAACGTGAAGTGTCAGGAG R: AATGCGTTCCTCAACCTGTG		211	
	TspE4.C2	F: CACTATTCGTAAGGTCATCC R: AGTTTATCGCTGCGGGTCGC		152	
	*arpA*	F: AACGCTATTCGCCAGCTTGC R: TCTCCCCATACCGTACGCTA		400	
Group E	*arpA*	F: GATTCCATCTTGTCAAAATATGCC R: GAAAAGAAAAAGAATTCCCAAGAG	57	219	
Group C	*trpA*	F: AGTTTTATGCCCAGTGCGAG R: TCTGCGCCGGTCACGCCC	59	489	
β- Lactamase/ESBL genes					
Triplex	*Bla*_TEM_	F: CATTTCCGTGTCGCCCTTATTC R: CGTTCATCCATAGTTGCCTGAC	57	800	[50]
	*Bla*_SHV_	F: AGCCGCTTGAGCAAATTAAAC R: ATCCCGCAGATAAATCACCAC		713	
	*Bla*_OXA_	F: GGCACCAGATTCAACTTTCAAG R: GACCCCAAGTTTCCTGTAAGTG		564	
Uniplex	*Bla*_CTX-M_	F: ATGTGCAGYACCAGTAARGTKATGGC R: TGGGTRAARTARGTSACCAGAAYCAGCGG	61	593	[51]
APEC virulence genotyping					
*iroN*		F: AATCCGGCAAAGAGACGAACCGCCT R: GTTCGGGCAACCCCTGCTTTGACTTT	60	553	[5]
*ompT*		F: TCATCCCGGAAGCCTCCCTCACTACTAT R: TAGCGTTTGCTGCACTGGCTTCTGATAC		496	
*hlyF*		F: GGCCACAGTCGTTTAGGGTGCTTACC R: GGCGGTTTAGGCATTCCGATACTCAG		450	
*iss*		F: CAGCAACCCGAACCACTTGATG R: AGCATTGCCAGAGCGGCAGAA		323	
*iutA*		F: GGCTGGACATCATGGGAACTGG R: CGTCGGGAACGGGTAGAATCG		302	
Plasmid replicon typing					
1	B/O	F: GCGGTCCGGAAAGCCAGAAAAC R: TCTGCGTTCCGCCAAGTTCGA	60	159	[35]
	FIC	F: GTGAACTGGCAGATGAGGAAGG R: TTCTCCTCGTCGCCAAACTAGAT		262	
	A/C	F: GAGAACCAAAGACAAAGACCTGGA R: ACGACAAACCTGAATTGCCTCCTT		465	
	P	F: CTATGGCCCTGCAAACGCGCCAGAAA R: TCACGCGCCAGGGCGCAGCC		534	
	T	F: TTGGCCTGTTTGTGCCTAAACCAT R: CGTTGATTACACTTAGCTTTGGAC		750	
2	K/B	F: GCGGTCCGGAAAGCCAGAAAAC R: TCTTTCACGAGCCCGCCAAA	60	160	
	W	F: CCTAAGAACAACAAAGCCCCCG R: GGTGCGCGGCATAGAACCGT		242	
	FIIA	F: CTGTCGTAAGCTGATGGC R: CTCTGCCACAAACTTCAGC		270	
	FIA	F: CCATGCTGGTTCTAGAGAAGGTG R: GTATATCCTTACTGGCTTCCGCAG		462	
	FIB	F: GGAGTTCTGACACACGATTTTCTG R: CTCCCGTCGCTTCAGGGCATT		702	
	Y	F: AATTCAAACAACACTGTGCAGCCTG R: GCGAGAATGGACGATTACAAAACTTT		765	
3	I1	F: CGAAAGCCGGACGGCAGAA R: TCGTCGTTCCGCCAAGTTCGT	60	139	
	Frep	F: TGATCGTTTAAGGAATTTTG R: GAAGATCAGTCACACCATCC		270	
	X	F: AACCTTAGAGGCTATTTAAGTTGCTGAT R: TGAGAGTCAATTTTTATCTCATGTTTTAGC		376	
	HI1	F: GGAGCGATGGATTACTTCAGTAC R: TGCCGTTTCACCTCGTGAGTA		471	
	N	F: GTCTAACGAGCTTACCGAAG R: GTTTCAACTCTGCCAAGTTC		559	
	HI2	F: TTTCTCCTGAGTCACCTGTTAACAC R: GGCTCACTACCGTTGTCATCCT		644	
	L/M	F: GGATGAAAACTATCAGCATCTGAAG R: CTGCAGGGGCGATTCTTTAGG		785	

### Phylogenetic groups

Phylogenetic groups of the isolates were determined using the updated quadruplex-PCR method developed by Clermont et al., when necessary, additional PCRs were applied to determine the phylotypes as recommended [23]. This method is capable of assigning *E. coli* isolate to one of the eight phylogenetic groups: A, B1, B2, C, D, E, F, clade I. Moreover, it allows isolates that are members of other cryptic clades (II to V) of *Escherichia* to be identified.

Each PCR reactions was performed in a volume of 20 μl containing: 10 μl *Taq* DNA Polymerase Master Mix RED 2x (Amplicon, Denmark) containing 1.5 mM MgCl_2_, various concentration of each primers, ultrapure water and 300 ng of template DNA. Primer characteristics and thermal conditions are shown in Table 5. Finally, PCR products were analyzed by electrophoresis using 1.5% (w/v) agarose gel and Green Viewer safe stain (0.01 v/v).

### APEC virulence genotyping

A total of 72 isolates were screened for five virulence genes (*iroN*, *ompT*, *hlyF*, *iss*, *iutA*) known as minimal predictors of APEC virulence [5]. Each PCR reactions was performed in a volume of 20 μl containing: 10 μl *Taq* DNA Polymerase Master Mix RED 2x (Amplicon, Denmark) containing 1.5 mM MgCl_2_, 1 μl of each forward and reverse primers, ultrapure water and 300 ng of template DNA. Primer characteristics and thermal conditions are shown in Table 5. Finally, PCR products were analyzed by electrophoresis using 1.5% (w/v) agarose gel and Green Viewer safe stain (0.01 v/v).

### Plasmid replicon typing

Plasmid replicon types of the APEC isolates were defined using the method designed by Johnson et al. [35]. This is a simplified method which requires only three multiplex-PCR panels to identify 18 plasmid replicons. The panels are as follow: panel 1: (B/O, FIC, A/C, P, T); panel 2: (K/B, W, FIIA, FIA, FIB, Y); panel 3: (I1, Frep, X, HI1, N, HI2, L/M).

Each PCR reactions was performed in a volume of 20 μl containing: 10 μl *Taq* DNA Polymerase Master Mix RED 2x (Amplicon, Denmark) containing 1.5 mM MgCl_2_, various concentration of each primers, ultrapure water and 300 ng of template DNA. Primer characteristics are shown in Table 5. Thermal conditions of PCR reactions were as follows: 5 min at 94 °C; 30 cycles of 30 s at 94 °C, 30s at 60 °C, and 90s at 72 °C; and a final extension of 5 min at 72 °C. Lastly, PCR products were visualized by electrophoresis using 1.5% (w/v) agarose gel and Green Viewer safe stain (0.01 v/v).

### Statistical analysis and data visualization

In addition to descriptive review of the results, possible relations among genetic criteria (phylogenetic groups, VGs, replicon types, ESBL genes) with phenotypic AMR were determined by chi-square test and Fisher’s exact test. In cases of significant associations (*p* < 0.05), correlations were determined using Spearman’s correlation coefficient. In the present study, calculations were performed in SPSS version 16.0 (SPSS Inc., Chicago, USA).

For better ilustration of the results, cluster analysis was performed (UPGMA method) using online tool CIMminer and a heatmap was also generated (http://discover.nci.nih.gov/cimminer).

## Supplementary Information

**Additional file 1: Supplementary 1.** Correlation coefficients for significant associations (*p* < 0.05) among genomic criteria (virulence types, phylotypes, plasmid types) with AMR (phenotypic and ESBL genes).

## Data Availability

Most data generated or analyzed during the current study are included in this published article. The other data are available from the corresponding author on reasonable request.

## Abbreviations

APEC: Avian pathogenic *Escherichia coli*
AMR: Antimicrobial resistance
MDR: Multidrug-resistant
HGT: Horizontal gene transfer
ESBL: Extended-spectrum beta-lactamase
Inc: Plasmid incompatibility
PBRT: PCR-Based Replicon Typing
VAG: Virulence-associated genes
VS: Virulence scores
AMC: Amoxicillin-clavulanic acid
TET: Tetracycline
NEO: Neomycin
FLO: Florfenicol
ENFX: Enrofloxacin
GM: Gentamicin
SXT: Trimethoprim-sulfamethoxazole
CST: Colistin
CFZ: Cefazolin
FDZ: Furazolidone
